# Excepting *Myotis capaccinii*, the wings' contribution to take-off performance does not correlate with foraging ecology in six species of insectivorous bat

**DOI:** 10.1242/bio.20149159

**Published:** 2014-10-17

**Authors:** James D. Gardiner, John D. Altringham, Elena Papadatou, Robert L. Nudds

**Affiliations:** 1 School of Computing, Science and Engineering, University of Salford, Salford M5 4WT, UK; 2 The School of Biology, University of Leeds, Leeds LS2 9JT, UK; 3 Faculty of Life Sciences, University of Manchester, Manchester M13 9PT, UK

**Keywords:** biomechanics, jumping, muscle, scaling, take-off, bat

## Abstract

Take-off in bats is separated into two distinct phases: an initial jump and a subsequent wing powered acceleration. Here, using footage from a high-speed camera, the first comparative study of the performance during the wing induced phase of take-off in six insectivorous bat species is described. Despite distinct differences in foraging strategy, the mass specific power generated by the bats during wing induced take-off did not differ between species, with the exception of *Myotis capaccinii*. This suggests that differences in take-off performance may only be evident in bats that exhibit particularly unusual foraging strategies, such as the trawling behaviour of *M. capaccinii* – with differences in the remaining species only manifesting in subtler aspects of flight performance such as agility or manoeuvrability. The poorer take-off performance of *M. capaccinii* could be related to either a reduction in wing-stroke amplitude to stop the wings hitting the water's surface during foraging or perhaps an effect of having very large feet. No scaling relationship between body mass and mass-specific take-off power was found, which supports earlier research on birds and insects, suggesting that the mass-specific muscle power available for flight is broadly similar across a large range of body sizes and species.

## INTRODUCTION

The ability to accelerate from slow flight, hovering or perching to a faster speed is a fundamental requirement for all flying animals. The acceleration may be provided passively by simply dropping from a perch or gaining lift from an oncoming breeze. In most cases, however, the acceleration is achieved by active flapping of the wings. The ability to take-off rapidly may have profound consequences for survival by, for example, reducing the chances of predation (Cresswell, 1993). Alternatively, slowing down and then accelerating back to cruising speed may be essential for some prey capture strategies such as gleaning from both vegetation and water, or for negotiating cluttered environments. Several studies have focussed on take-off performance and accelerating flight in birds (Askew et al., 2001; Berg and Biewener, 2010; Earls, 2000; Jackson and Dial, 2011; Tobalske and Dial, 2000; Tobalske et al., 2004), but far fewer studies have examined bat take-off (Altenbach, 1979; Gardiner and Nudds, 2011; Schutt et al., 1997). In contrast to birds where the wings only play an aerodynamic role, bat wings have two separate functions during a two-phase take-off (Gardiner and Nudds, 2011). The initial jump phase using the wings as levers to jump off the ground (which has been the focus of studies so far) and then the accelerating flight phase using the wings as aerodynamic surfaces (the focus of this study). We will term the second aerodynamic phase, ‘wing induced take-off’ and it is defined here as take-off excluding the momentum provided by the legs (birds), or forearms (bats), during the preceding flight-initiating jump. The separation point between the two phases is defined as the start of the first down-stroke of the bat's wing.

Gardiner and Nudds showed that contrary to expectations the jump performance of gleaning bats (i.e. bats that forage on or near the ground) was not significantly better than hawking bats (i.e. bats that catch prey on the wing) (Gardiner and Nudds, 2011). Bats use their wings as levers to catapult themselves into the air when taking-off from the ground and wingspan (*b*) in flying vertebrates is tightly constrained by aerodynamic requirements to a geometric scaling with body mass i.e. *M*_b_^1/3^ (Norberg, 1990; Norberg and Rayner, 1987; Nudds, 2007; Rayner, 1988). Hence, the fact that the levers bats use to catapult themselves into the air are similar among species, coupled with the fact that a bat's jumping ability is derivative, being enabled by the strongly selected for flight apparatus, may explain the finding that mass-specific jump performance in five species of bats was similar with no ecological trend apparent (Gardiner and Nudds, 2011). In contrast to wingspan (*b*), there is considerable variation in other features of bat wing-morphology (e.g. wing area and aspect ratio) related to their ecology (Norberg and Rayner, 1987). Therefore, unlike flight initiating jumps, maximum (aerodynamic) forces and hence the power generated by the wings may differ substantially among species of bat. The power a bat can invest in moving its centre of mass (i.e. the power in excess of the minimum aerodynamic requirements) is a good indicator of its overall wing induced take-off performance.

The first aim of this current study was to calculate the wing induced take-off performance of six species of bat (*Miniopterus schreibersii*, *Myotis blythii*, *Myotis capaccinii*, *Myotis myotis*, *Rhinolophus blasii* and *Rhinolophus euryale*), after they have completed their initial jump from the ground. Indeed, the jump performance of the same study bats (excluding *Rhinolophus euryale*) was previously reported by Gardiner and Nudds (Gardiner and Nudds, 2011), but please note that none of the wing induced take-off performance data has been published previously. *Miniopterus schreibersii* is an aerial hawker feeding in open areas at relatively high altitudes (Norberg and Rayner, 1987). Depending on food availability, the sister species *M. blythii* and *M. myotis* both have flexible foraging behaviour, which includes ground gleaning and aerial hawking (Arlettaz, 1996). *Myotis capaccinii* has a highly specialised foraging behaviour trawling and gaffing insects and small fish from water surfaces (Aihartza et al., 2008; Aizpurua et al., 2013). The horseshoe bats *R. blasii* and *R. euryale* typically forage as aerial hawkers in cluttered woodlands. Additionally *R. blasii* has been observed to glean from the ground, sometimes even chasing prey (Siemers and Ivanova, 2004). Intuitively, it seems likely that all of the flight apparatus (muscle and wing morphology) would be tuned to the flight requirements of the species, with the result manifesting as the power generated by the wings. Therefore, species that often need to fly slowly when foraging should have a better take-off performance than those species that conduct their foraging at higher speeds.

The second aim was to investigate the scaling of power available from bat flight muscles. In general, mass-specific power is predicted to increase more slowly with *M*_b_ than the power required for flight (Norberg and Norberg, 2012; Pennycuick, 1968). Hence, a decrease of additional power available for manoeuvres and accelerations, and, therefore, take-off performance, is expected as animal size increases. Indeed, Tobalske and Dial showed that in the Phasianidae (Aves) this excess power scaled against *M*_b_ with an exponent of 0.68 (Tobalske and Dial, 2000), which meant that as birds got larger the mass-specific take-off power available for accelerating flight decreased. Pectoralis muscle mass-specific power during take-off scales negatively with *M*_b_: scaling as *M*_b_^−1/3^ in the Phasianidae (Tobalske and Dial, 2000) and *M*_b_^−0.18^ in the Corvidae (Jackson and Dial, 2011). In contrast, however, Askew et al. showed that pectoralis muscle mass-specific power across a large range of birds and bees was independent of *M*_b_ with all animals producing between 200–400 W/kg of flight muscle (Askew et al., 2001). Similarly, the mass-specific force generated by bats during their flight initiating jumps did not vary with *M*_b_ (Gardiner and Nudds, 2011).

Here, the relationship between the mass-specific power generated during wing induced take-off and body mass was investigated. Additionally, the hypothesis that the bats that tend to feed at lower flight speeds (i.e. the gleaners) will produce more mass-specific power during wing induced take-off than aerial hawkers, which are specialised for faster flight, was tested. Explicitly, mass specific power generated by the bats during wing induced take-off was hypothesised to fall into ranks depending on bat foraging strategy (Table 1).

**Table 1.**
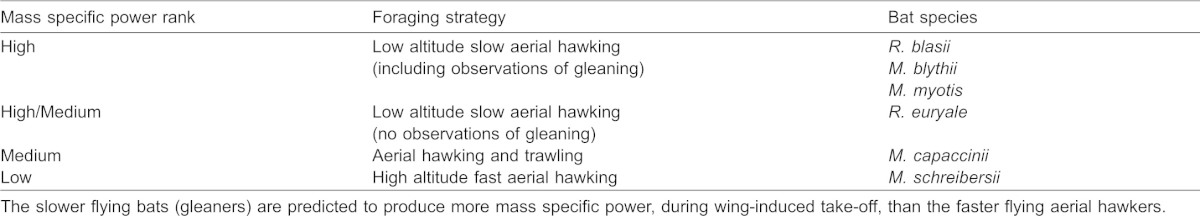
Hypothesised mass specific power ranking based on foraging strategy for the study bats

## MATERIALS AND METHODS

### Capturing bat flight footage

The bats were captured over three evenings (24/08/06, 25/08/06 and 25/08/07) using a harp trap positioned across the entrance of Polyphimos Cave near Maronia, Rhodope Prefecture, Greece. The *M*_b_ of each bat was measured prior to being released from a level platform (±5°). The handler held the bats upon the platform under a gloved hand with the head oriented in the desired take-off direction (left to right relative to the camera lens), which was toward the roosting cave. Once the bat was correctly positioned, the handler swiftly withdrew their hand and the bats were allowed to take-off. It was assumed that escaping from the handler elicited a maximum effort take-off. A white sheet hung behind the release platform ensured the bats flew perpendicular to the camera view and also made the bats more discernable in the footage. The take-off platform and white sheet were illuminated with floodlights (Nightsearcher, Portsmouth, UK). The flights were filmed at either 125 or 250 frames per second (fps) using a Fastec Trouble Shooter camera (Fastec Imaging, San Diego, CA, USA).

### Analysis of flight footage

Video footage of the bats accelerating away from the release platform was digitised using Tracker 3.10 (Open Source Physics): *M. blythii* (*n* = 3), *M. capaccinii* (*n* = 15), *M. myotis* (*n* = 5), *R. blasii* (*n* = 7), *R. euryale* (*n* = 2) and *M. schreibersii* (*n* = 17). Any footage in which the bat flew towards or away from the camera was disregarded. The images were scaled using a checkerboard and a plumb line allowed the images to be rotated so that the *x* and *y* axes of the frames corresponded to the horizontal and vertical respectively. Positional data were extracted from the start of the first down-stroke to the start of the fourth down-stroke (i.e. the first three complete wing-strokes). To estimate the position of the centre of mass (COM) of the bat during the take-off flight, the shoulder joint was tracked. Tracking the shoulder joint as a proxy for COM is not ideal, since the COM is known to vary in location during locomotion (Iriarte-Díaz and Swartz, 2008; Iriarte-Díaz et al., 2011). This method, however, is not likely to significantly affect our data analyses here, particularly with respect to the mean power, which is averaged over all three wing-strokes. The wing is in the same relative wing-stroke position (the very top) at the beginning and end of the video clips analysed.

### Data analyses

**Fig. 1.**
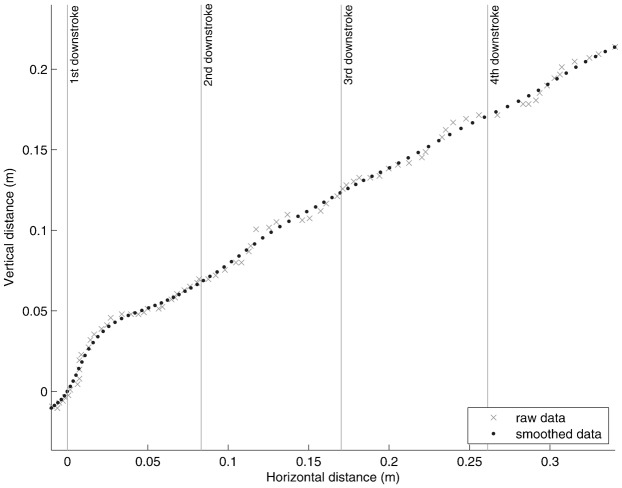
Wing induced take-off flight data from a *M. schreibersii*, showing the horizontal and vertical displacement of a bat from the digitised high-speed footage. Raw data (dots) and smoothed data (crosses), using a fourth order Butterworth low pass filter with a cut-off frequency of 14 Hz, are both shown.

The data collected from the videos were analysed using MATLAB® R2009a (The MathWorks, Inc., 3 Apple Hill Drive, Natick, MA). To smooth the raw data (Fig. 1) high frequency noise associated with digitisation errors was removed using a low-pass 4^th^ order Butterworth filter (cut-off 14Hz). The cut-off frequency for the filter was chosen using the method described on page 42 of Winter, which selects the frequency with the best balance between noise-reduction and signal distortion, using plots of the residuals (i.e. a measure of the difference between the smoothed data and the raw data) (Winter, 1990). The smoothed data were then used to calculate the power associated with the movement of the COM. The power associated with the movement of the COM does not take into account the aerodynamic power the bat must also produce i.e. there is a minimum requirement for lift to match body weight and thrust to equal drag. Power above this minimum aerodynamic requirement is what is seen as the acceleration of the COM. Calculating this minimum aerodynamic power is difficult, requires many assumptions to be made and is beyond the scope of this study. Furthermore, from the bat's perspective the ability to move the COM during take-off (i.e. to escape potential predation threats or improve foraging success) is the critical measure of the acceleration performance. The take-off power was, therefore, calculated as the change in kinetic and potential energy over the three wing-strokes as
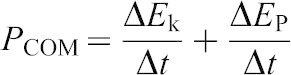
(1)where *P*_COM_ is the power (W) of the COM, Δ*E*_k_ is the change in kinetic energy (J), Δ*E*_p_ is the change in potential energy (J) and Δ*t* is the change in time (s). The change in kinetic energy was calculated as
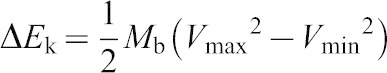
(2)where *M*_b_ is the body mass (kg), and *V*_min_ and *V*_max_ are the speed at the start of the first wing-stroke and end of the third wing-stroke respectively. The change in potential energy was calculated as
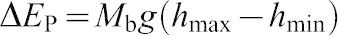
(3)where *g* is the acceleration due to gravity (ms^−2^), and *h*_min_ and *h*_max_ are the bat's height at the start of the first wing-stroke and end of the third wing-stroke respectively. The take-off power was divided by *M*_b_ to give the mass-specific take-off power (W/kg). Mean power, however, does not take into account fluctuations in the instantaneous power, which occur during each wing-stroke. ANOVA with a Tukey post hoc test was used to compare the mass-specific take-off power (W/kg) between the bat species. The scaling relationships of the mass-specific take-off power and wing-stroke frequency (Hz) against *M*_b_ were determined using ordinary least squares regressions. The latter as a metric of the effort each species was putting into power generation. Ordinary least squares regressions were chosen over reduced major axis regressions because the error in the *M*_b_ of the bats is likely to be significantly smaller than the error in the power calculations. All means are expressed ± standard error.

## RESULTS

The mean body masses (g) of the five species of bat were 25.70±0.93 (*M. blythii*), 8.69±0.42 (*M. capaccinii*), 26.78±0.72 (*M. myotis*), 10.51±0.61 (*R. blasii*), 9.9±1.14 (*R. euryale*) and 11.59±0.40 (*M. schreibersii*).

**Fig. 2.**
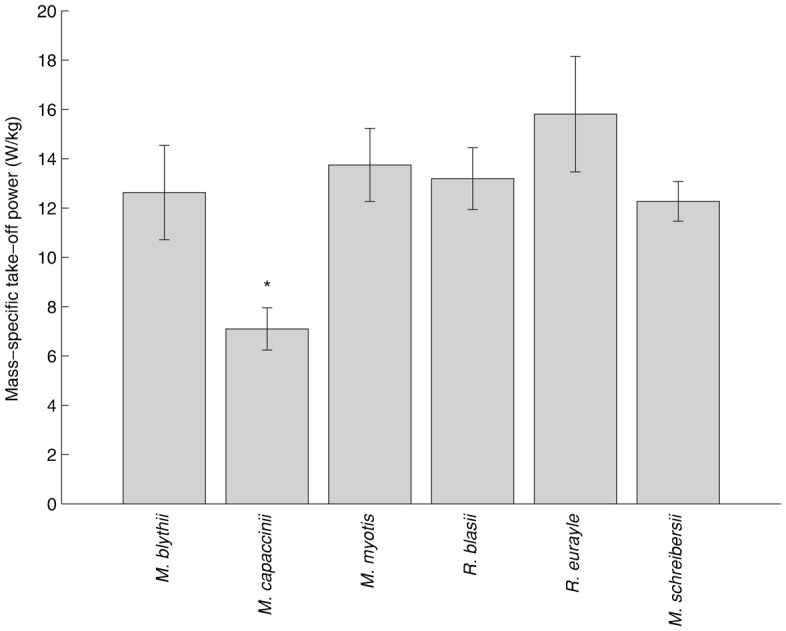
Mean mass-specific wing induced take-off power of six bat species: *M. blythii* (*n* = 3), *M. capaccinii* (*n* = 15), *M. myotis* (*n* = 5), *R. blasii* (*n* = 7), *R. euryale* (*n* = 2) and *M. schreibersii* (*n* = 17). Bars with the asterisk (*) are significantly different from others (ANOVA with Tukey's least significant difference (LSD) post-hoc procedure). All data are shown with standard error bars.

The mean mass-specific wing induced take-off power output (Fig. 2) differed between species (*F*_5,43_ = 6.92, *r*^2^ = 0.45, *p*<0.001). All bats except for *M. capaccinii*, however, produced between 12 and 16 W/kg of mass-specific power. *Myotis capaccinii* produced significantly lower mass-specific wing induced take-off power than all other species (Table 2). The mean values (W/kg) were 12.62±1.92 (*M. blythii*), 7.09±0.86 (*M. capaccinii*), 13.75±1.48 (*M. myotis*), 13.19±1.25 (*R. blasii*), 15.81±2.35 (*R. eurayle*) and 12.27±0.80 (*M. schreibersii*).

**Table 2.**
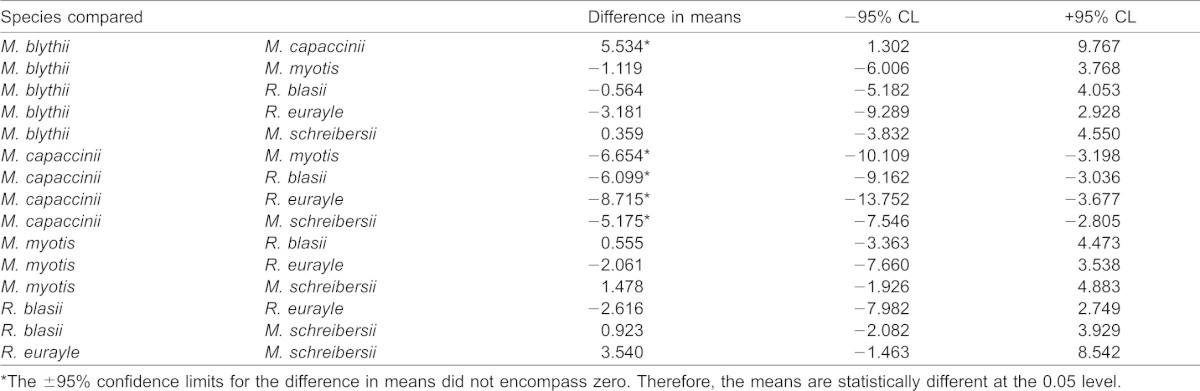
Mass specific wing induced take-off power pairwise comparison results using Tukey's least significant difference (LSD) post-hoc procedure

**Fig. 3.**
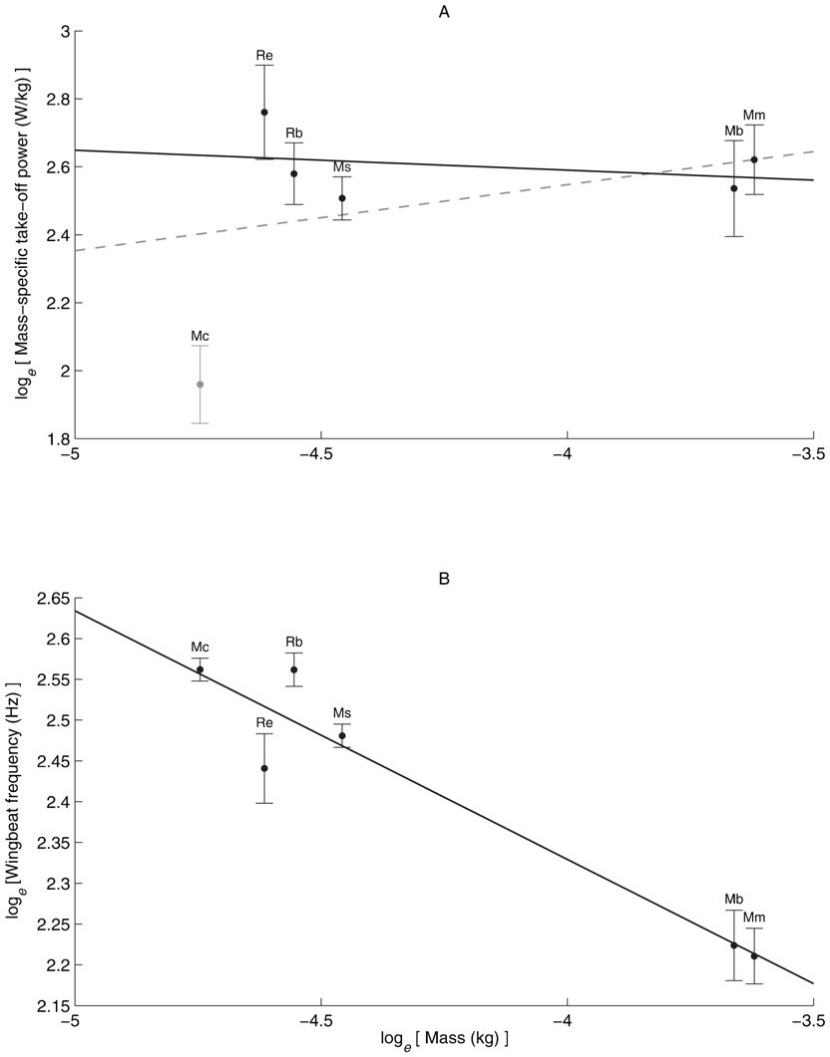
Scaling relationships for the six bat species *M. blythii* (Mb), *M. capaccinii* (Mc), *M. myotis* (Mm), *R. blasii* (Rb), *R. euryale* (Re) and *M. schreibersii* (Ms). (A) Showing no scaling relationship of log*_e_* mass-specific wing induced take-off power against log*_e_* body mass. Grey dashed line is scaling relationship including *M. capaccinii* (grey data point) and solid black line is relationship excluding *M. capaccinii*. (B) Scaling relationship of log*_e_* wing-beat frequency against log*_e_* body mass. Wing-beat frequency scaled (*y* = 1.11−0.305*x*) predictably against body mass. The slope of the relationship was not significantly different from the expected slope of −0.33.

Mean mass-specific wing induced take-off power was independent of *M*_b_ (*F*_1,4_ = 0.56, *r*^2^ = 0.12, *p* = 0.49) (Fig. 3A). When *M. capaccinii* was excluded from the regression (Fig. 3A), because it was an outlier, there was still no scaling relationship between mass-specific wing induced take-off power and *M*_b_ (*F*_1,3_ = 0.28, *r*^2^ = 0.09, *p* = 0.63).

Wing beat frequency (Fig. 3B) scaled negatively as *M*_b_^−0.305±0.045^ (*F*_1,4_ = 46.59, *r*^2^ = 0.92, *p* = 0.002) and the scaling exponent did not differ (*t*_4_ = 0.556, *p* = 0.61) from the expected exponent of *M*_b_^−0.33^ (Tobalske and Dial, 2000). The mean wing-beat frequencies (Hz) for each species were 9.24±0.41 (*M. blythii*), 12.96±0.18 (*M. capaccinii*), 9.12±0.32 (*M. myotis*), 12.96±0.27 (*R. blasii*), 11.48±0.50 (*R. eurayle*) and 11.95±0.17 (*M. schreibersii*).

## DISCUSSION

Contrary to our hypothesis, the mass-specific wing induced take-off power produced by the bats did not appear to correlate with foraging strategy (Fig. 2). Indeed, with the exception of *M. capacinnii* there was no significant difference between the mass-specific take-off power of the study bats despite variations in foraging strategies. For example *R. blasii* and *M. schreibersii*, whilst sharing similar body masses (10.51±0.61 and 11.59±0.40 respectively), have very different foraging strategies (gleaning and hawking amongst vegetation, versus high altitude high speed hawking) yet no significant difference was detected in their take-off power. This is a surprise since it is well documented that foraging strategy is strongly correlated to bat wing morphology (Norberg and Rayner, 1987), which in turn affects flight performance (Norberg, 1990; Rayner, 1988). It may be, however, that take-off performance does not exert strong selection pressure and differences in wing morphology only have a measurable influence on subtler aspects of flight performance, such as manoeuvrability and agility, or top speed. The fact that only *M. capaccinii* produced less take-off power than the other bats may be because of its highly specialised foraging strategy. *M. capaccinii* fishes and catches insects of the surface of the water (Aihartza et al., 2008). Large feet, which aid the gaffing of prey, may create a large moment of inertia around the bats centre of mass hindering a fast take-off flight. Additionally, although neither were measured here, a smaller relative pectoralis muscle mass, or perhaps a reduced wing-stroke amplitude could also affect mass-specific power production, with the latter perhaps being linked to the need to avoid dipping wings when foraging close to the water's surface. The fact that *M. capaccinii* was previously shown to perform as well as other species of bats during the first phase of take-off (flight-initiating jump performance) (Gardiner and Nudds, 2011), however, suggests that a relatively small pectoralis is unlikely. The wing-beat frequency of all bats scaled as predicted and *M. capaccinii* did not appear to be an outlier, so the result cannot be explained by lower wing-beat frequency and hence power in *M. capaccinii*. The wingspan, forearm length and wing loading of *M. capaccinii* relative to its *M*_b_ are also not exceptional, with the lengths being similar to all the other five bats in this study and the wing loading similar to the other *Myotis* species and *M. schreibersii* (Gardiner and Nudds, 2011; Norberg and Rayner, 1987).

The mass-specific power of the bats did not scale negatively with body mass as we hypothesised. In fact, both with *M. capaccinii* included and excluded, there was no significant scaling relationship between mass specific take-off power and body mass. The absence of a scaling relationship between take-off power and mass reflects the conclusion drawn by Askew et al. for the power output of flight muscles for insects and birds (Askew et al., 2001). They showed that over a body mass range of 170 mg to 4.78 kg, and despite large variations in flight style and wing-beat frequency, all the study animals produced similar mass-specific flight muscle power. It should be noted, however, that the bat data here are from only three bat families, which allows the possibility that phylogenetic effects have influenced the scaling relationships. The distribution of data (Fig. 3), however, suggests that phylogeny has not confounded the mass specific take-off power scaling relationship, with the data points for both *Rhinolophid* species and the two large *Myotis* species falling closely together respectively. Furthermore, since no scaling relationships were found between mass-specific wing induced take-off power and body mass (both with *M. capacinni* included and excluded) it is unlikely a phylogenetically corrected analysis will find a scaling relationship, since these techniques are generally more conservative.

In this article, to the best of our knowledge, we present the first comparative study in bats of the take-off flight power contributed by the wings only (i.e. minus the jump phase). Other studies, however, have tackled different aspects of bat take-off, which help improve our overall understanding of vertebrate flight and aerodynamics. A recent study by Adams et al. suggested that the tail membrane of bats may also play and important role in bat take-off by providing additional thrust (Adams et al., 2012). The authors noted that the bat's tail membrane was flapped during take-off in a manner consistent with expectations for an aerofoil. The tail membranes of our study bats (including the outlier *M. capacinnii*) are morphologically similar. Nonetheless, differences in tail membrane kinematics may have contributed to the low mass-specific power produced by *M. capacinnii*. Additionally, both wing shape and wing tension may be controlled throughout flight, by the hind legs (Cheney et al., 2014a) and also small muscles embedded within the wing membrane (Cheney et al., 2014b), increasing the range of flight modes (potentially including take-off) where bats are aerodynamically efficient. How the use of the legs and membrane muscles affects take-off performance and accelerating flight, however, remains unknown.

In summary, our study bats show that differences in take-off/escape flight performance may only be detectable in bat species with more extraordinary foraging strategies like *M. capaccinii* (in this study) or perhaps other specialist species like the vampire bats, *Desmodus* (Schutt et al., 1997). Furthermore, any differences will likely be due to morphology and not because of size dependent differences in mass-specific power, particularly as evidence against this hypothesis is building.
